
JOURNAL INFORMATION
==============================
NLM Title Abbreviation: Buffalo Med J Mon Rev Med Surg Sci
NLM Journal ID: 0004276

The Buffalo Medical Journal and Monthly Review of Medical and Surgical Science

Publisher: Biomedical Journal Digitization Project

ARTICLE INFORMATION
==============================
PMCID: PMC8932452
PMID: 35376949
Article version: 1
Subjects: Eclectic Department

A Case of Obstruction of the Bowels relieved by Copious Injections, after the Failure of other means

Print publication date: 1858 Dec
Volume: 14
Issue: 7
First page: 433
Last page: 434
License: This work is free of known copyright restrictions. For more information, please see PMC Back Issue Digitization.
License URL: https://creativecommons.org/publicdomain/mark/1.0/

==============================

### *A Case of Obstruction of the Bowels relieved by Copious Injections, after the Failure of other means.* By O. C. Gibbs, M. D.

The following case is interesting, because relief came when but little expected, and under desperate circumstances:

*January* 1*st*, 1858.—I was called to see Mrs R———, aged about 55 years. She complained of severe pain in the bowels, was vomiting some, the pulse was quiet, tongue furred, bowels tender on pressure, the countenance haggard and indicative of much distress and prostration. I suspected a a strangulated hernia, but, on inquiry and examination, this supposition proved groundless. Opium, in full doses, combined with small doses of calomel, was advised internally, and hot fomentations locally.

*January* 2*d.*—The patient was suffering less pain, but not otherwise improved. The treatment was continued, the opium in diminished, and the calomel in increased, doses.

*January* 3*d.*—The patient was no better; the pulse was more frequen and the vomiting still continued. Castor oil was now ordered in tablespoonful doses, to be repeated every hour until it operated; the action of the oil to be aided by injections of infusion of senna.

*January* 4*th.*—Still no improvement, but patient gradually failing; Bowels were much bloated and tympanitic. The matters vomited now were stercoraceous. Cloths, saturated in turpentine, were applied to the bowels. calomel, rubbed up with blue-pill, was now ordered, in four grain doses of the mass, to be repeated every two hours.

*January* 5*th.*—Treatment had been discontinued during the night; the friend supposed the patient dying, and refused to give additional medicines. The patient was evidently sinking; the bowels were greatly distended, the vomiting stercoraceous, no operation upon the bowels had yet been secured. Though the case was unpromising, I urged the friends to additional efforts. A blister was ordered over the stomach; as soon as drawn, the cuticle was directed to be removed, and one grain of morphine to be applied to the denuded surface; turpentine was still ordered to the bowels; and calomel, in five grain doses, to be administered every three hours.

*January* 6*th.*—Still no operation from the bowels, and no cessation of the vomiting. The patient was slowly sinking. Ordered treatment continued, though, I must confess, with no hopes of relief.

*January* 7*th.*—The patient was supposed to be dying by friends; medicines had been discontinued since 12 o'clock last night.

I now explained to the friends that it was possible that a copious injection might overcome the obstruction, and afford relief; this, I said, will occasion much pain, but we certainly ought not to let the patient die without making at least one more effort to afford relief. Of the many present, I selected two women of nerve and decision, to carry out my directions. With a pump, I ordered them to inject tepid water into the bowels, so long as they could prevail upon the patient to endure it. This they did, and returned to me soon, informing me that they had injected only about a pint. The patient's sufferings, they said, were extreme. Less had been accomplished than I had expected.

I now took the pump myself, closed the door against spectators, and commenced injecting, entreating the patient to endure to the utmost, as this was her only hope, This she did, for a time, but soon her shrieks and groans became heart-rending. Her husband and son now rushed into the room, and commanded me to desist from further attempts to relieve the patient; which I did only after at least two quarts had been injected. The friends evidently looked upon me as a personification of brutality. Though conscious of having done my duty, I felt confident I had lost the confidence of the many present, and that I had better have let the patient died, at least without the last effort for relief.

About an hour after this, to my great joy, the bowels moved freely and repeatedly, to the no inconsiderable relief of the patient. From this time on, the only laxative required was a little yeast. The patient, however, was not destined to recover. She died about two weeks later, seemingly from an inability to rally from the extreme prostration. Peritoneal inflammation was, doubtless, the cause of death, which, in a person of less years, and under more favorable circumstances, might have been cured.

I can not avoid the conviction, that if the copious injections, with a view of forcing the obstruction, had been resorted to earlier, the result might have been different.

FREWSBURG, Chatauque Co., N. Y.

---

*Anæsthetics.*—We quote the following article from the Lancet for October 16, 1858, and we have no doubt that all it asserts is true. Indeed, we have looked over the late Dr. Snow's work with much interest, and can heartily join in the recommendation of the Lancet, to give this "valuable monograph" a "thoughtful perusal." Notwithstanding all that is said, however, even by the highest authority, we believe that the time is not far distant when chloroform will be abandoned as an anæsthetic agent. We again ask, why use an article of such uncontrollable power, when we have an equally reliable one in ether, and one which is entirely safe? It is notorious, that under the best conditions, and in the most careful hands, chloroform is a dangerous weapon. It is only lately that Mr. Erichsen, high surgical authority, has stated both pubilcly and privately, that he uses chloroform as he wonld take an *express train*, ignoring, or risking, the dangers of extra velocity. Now, is it not better to travel on the anæsthetic track a little more slowly, and be safe in our journey? Mr. Erichsen himself intimates as much as this; and the evidence of every day only confirms us in the opinions we have expressed.

"The frequent recurrences of death from the administration of chloroform during the last few weeks has induced a feeling of uneasiness and distrust of this agent both in the profession and out of it. It is very certain, however, that a great part of this feeling might very advantageously be transferred to the mode of administration which is still in vogue. It must not be forgotten that, in all these cases, the chloroform was administered loosely on a handkerchief, and in more than one instance, as has been proved, it was of a thickly-woven texture, which did not allow of a free passage of air. This mode of administration is, we think, most unjustifiable. The experiments of the late Dr. Snow conclusively showed the necessity of carefully regulating the proportion of vapor in the air inspired. We strongly recom-
